# Comparative genomic analysis of the zebra finch degradome provides new insights into evolution of proteases in birds and mammals

**DOI:** 10.1186/1471-2164-11-220

**Published:** 2010-04-01

**Authors:** Víctor Quesada, Gloria Velasco, Xose S Puente, Wesley C Warren, Carlos López-Otín

**Affiliations:** 1 Departamento de Bioquímica y Biología Molecular, Facultad de Medicina, Instituto Universitario de Oncología, Universidad de Oviedo, 33006-Oviedo, Spain; 2 Genome Sequencing Center, Washington University School of Medicine, St Louis, Missouri, USA

## Abstract

**Background:**

The degradome -the complete repertoire of proteases in an organism- is involved in multiple key biological and pathological processes. Previous studies in several organisms have yielded sets of curated protease sequences which may be used to characterize the degradome in a novel genome by similarity. Differences between degradomes can then be related to physiological traits of the species under study. Therefore, the sequencing of the zebra finch genome allows the comparison between the degradomes of mammals and birds and may help to understand the biological peculiarities of the zebra finch.

**Results:**

A set of curated protease sequences from humans and chicken was used to predict the sequences of 460 protease and protease-like genes in the zebra finch genome. This analysis revealed important differences in the evolution of mammalian and bird degradomes, including genomic expansions and deletions of caspases, cytotoxic proteases, kallikreins, matrix metalloproteases, and trypsin-like proteases. Furthermore, we found several zebra finch-specific features, such as duplications in *CASP3 *and *BACE*, and a large genomic expansion of acrosin.

**Conclusions:**

We have compared the degradomes of zebra finch, chicken and several mammalian species, with the finding of multiple differences which illustrate the evolution of the protease complement of these organisms. Detailed analysis of these changes in zebra finch proteases has shown that they are mainly related to immunological, developmental, reproductive and neural functions.

## Background

The degradome is defined as the set of proteases present in an organism [1]. The coining of this term reflects the enormous biological and pathological relevance of proteolysis that pervades virtually every aspect of life, including development, apoptosis, host defense, nutrition, reproduction and central nervous system biology [2]. From a genomic perspective, the degradome provides a relatively simple representative subset of the coding genome of a species. Thus, the human degradome contains about 570 proteases, and can be studied with semi-automated methods. On the other hand, and despite proteases share a common biochemical function, their catalytic domains exhibit high sequence diversity. This diversity is further increased by the frequent attachment of auxiliary, non-proteolytic domains to the catalytic moieties [3]. It is also remarkable that while some of the protease genes are clustered, most of them are randomly distributed throughout the annotated genomes [4-7]. Hence, the degradome forms a representative subset of the coding genome of a species, both in terms of sequence and genomic organization.

Furthermore, since the role of multiple proteases in biological processes is well documented, the comparative study of degradomes may improve our understanding of these processes in different organisms. The zebra finch (*Taeniopygia guttata*) is a bird which has been extensively used as a model organism for neurological [8], reproductive [9], and immune [10] studies. Therefore, the characterization of the zebra finch degradome may provide valuable information on the role of proteases in key biological processes.

In this work, we report the analysis of the complete set of protease and protease-like genes in the zebra finch genome and its comparison to the chicken and human degradomes. Where appropriate, other mammalian degradomes have also been considered to expand the scope of this protease-based comparative genomic analysis.

## Results and Discussion

As expected, we have found that the zebra finch degradome is similar to the chicken degradome. According to our preliminary prediction, the zebra finch and chicken degradomes contain about 460 proteases. Reciprocal best hit analysis of these results with the Ensembl predicted protein complement of chicken showed that 380 zebra finch proteases have a reciprocal best hit orthologue in the chicken degradome. In contrast, 80 predicted zebra finch proteases have no clear orthologue in chicken. Most of these proteases belong to large complex families, in which orthology assignment is difficult to establish. It must also be noted that gene gains and losses may be confused with lack of genomic data and assembly artefacts. However, some of these zebra finch specific proteases seem to have clearly arisen from species-specific duplications.

Comparison of these degradomes with the human degradome has yielded further information about the evolution of proteases and has led to hypothesis about their putative roles in zebra finch physiology. Thus, most of the major differences characteristic of the zebra finch and chicken degradomes compared to mammalian degradomes affect proteases involved in a few key biological processes, including apoptosis, host defense, teeth formation, reproduction, and neural development.

### Apoptosis

The detailed comparative analysis of proteases involved in apoptosis has revealed a series of zebra finch and avian characteristic features. Thus, group I caspases, involved in the processing of inflammatory cytokines, is composed of *CASP1*, *-4*, *-5*, and *-12 *in humans [11] and only one (*CASP1*) in chicken. We have not found any sequence corresponding to group I caspases in the current assembly of zebra finch nor in zebra finch ChrUn assembly or sequence traces, nor in EST databases. However, the genomic contig in which this sequence should be located is rich in unsequenced stretches. Since this protease is expected to play a very important role in apoptosis induced by bacterial infections, it seems likely that the gene encoding caspase-1 exists in an unsequenced stretch of the zebra finch genome. Nevertheless, further experimental validation will be necessary to clarify this important question.

**Figure 1:**
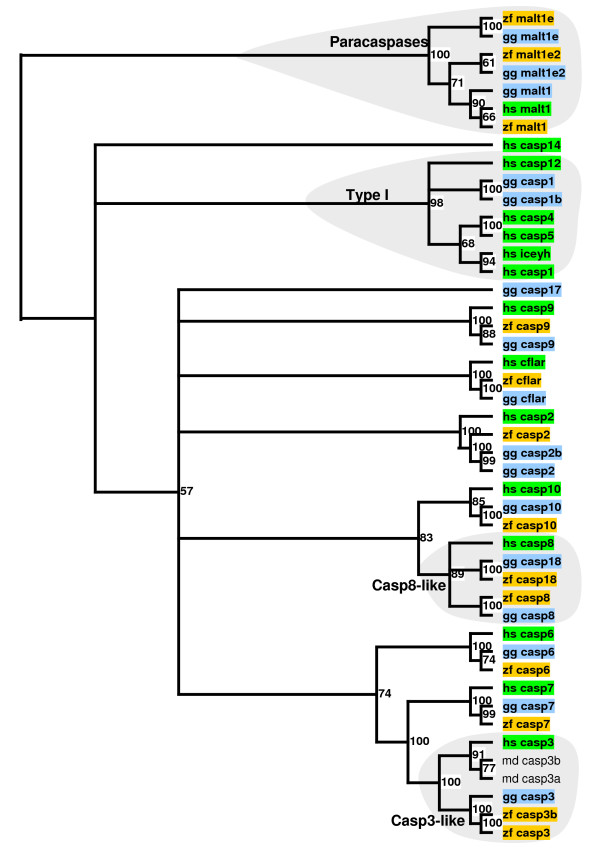
**Phylogenetic relationship between zebra finch (*zf_*), chicken (*gg_*), and human (*hs_*) caspases**. Numbers indicate the robustness of each node assessed by bootstrap. Two caspase-3-like genes from opossum (*md_*) are included. *Casp*, caspase; *malt*, paracaspase; *cflar*, casper; *iceyh*, homolog ICEY. The phylogenetic tree was rooted with the paracaspase family.

On the other hand, the degradomes of zebra finch and chicken contain several caspases which are not present in humans [12] (Figure 1). Thus, *CASP18 *is present in both birds. The phylogenetic analysis of this gene is consistent with the proposed evolutionary history of this gene, which is present in opossum and absent in placental mammals [12]. Another gene absent in mammals is *CASP17*, which can be found in chicken. Surprisingly, we have not found any orthologue of *CASP17 *in the zebra finch genome. In contrast, there is evidence for a tandem duplication of *CASP3 *in the zebra finch genome, but not in the genomes of chicken or human. Taken together, these results suggest that the mechanisms leading to caspase-dependent apoptosis in birds might depend on more proteases than in mammals. In fact, the evolution of caspases in mammals includes complex events, like the pseudogenization of *CASP12 *in most of the human population but not in other hominoids [13]. These changes may influence processes such as immune system maturation and inflammatory response. In this regard, it is noteworthy that recent data have indicated that blocking caspase-mediated apoptosis reduces neurogenesis in the song nucleus of a bird [14].

### Host defense

Proteases play important roles in the immune defense against pathogens by acting as cytotoxic agents or by contributing to processes of activation and proliferation of immune cells. The pressure of pathogens on the evolution of the immune system has likely led to marked differences in these proteases between birds and mammals.

**Figure 2:**
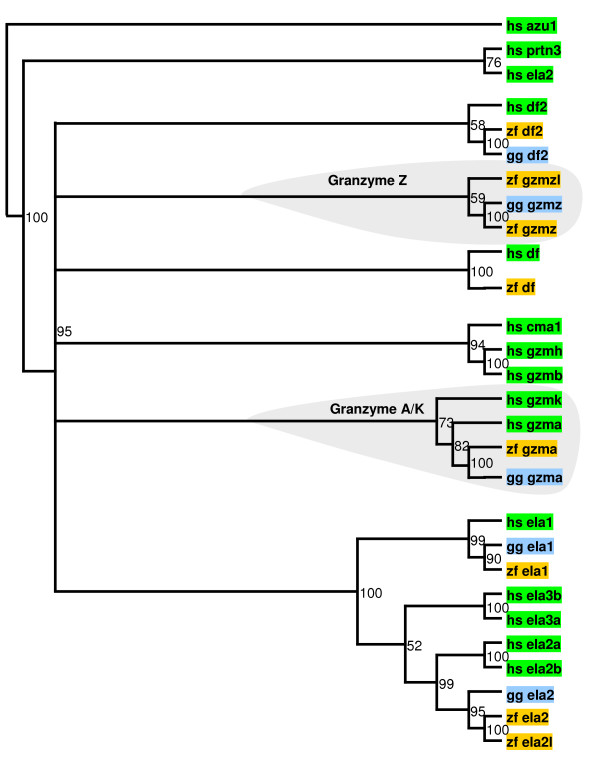
**Phylogenetic relationship between zebra finch (*zf_*), chicken (*gg_*), and human (*hs_*) proteases in granzyme clusters**. Numbers indicate the robustness of each node assessed by bootstrap. *Azu1*, azurocidin-1; *df2*, complement factor D; *ela2*, neutrophil elastase; *prtn3*, proteinase 3; *gzm*, granzyme; *ela*, elastase; *cma1*, chymase. Human azurocidin is included as an outgroup.

Immune cytotoxic proteases are serine proteases stored in the granules of neutrophils, mast cells, and natural killer lymphocytes and released upon activation. Once released, these proteases promote apoptosis of infected cells [15]. In humans, the genes encoding cytotoxic proteases are clustered in three genomic loci. The first cluster contains the genes encoding granzymes A (*GZMA*) and K (*GZMK*). As shown in Figure 2, *GZMK *is absent in the genomes of zebra finch and chicken, whereas *GZMA *is conserved in both birds. The second human cluster contains neutrophil elastase (*ELA2*), complement factor D (*DF*), azurocidin (*AZU1*), and proteinase 3 (*PRTN3*). This cluster seems to be lacking in both zebra finch and chicken (Figure 2). Finally, the third human cluster contains granzyme B (*GZMB*), granzyme H (*GZMH*), cathepsin G (*CTSG*), and chymase (*CMA1*). The genomes of both zebra finch and chicken contain a single protease gene related to all four human proteases, which we have named *GZMZ*. Notably, this protease has been duplicated in the zebra finch. The resulting novel zebra finch-specific granzyme (*GZMZL*) is classified as a non-peptidase homolog, lacking proteolytic capabilities, since its sequence features two substitutions at key catalytic residues. It should be noted that the most abundant granzymes in humans are A and B, which cause caspase-independent and caspase-dependent apoptosis, respectively. Therefore, this analysis predicts that avian granzyme A and granzyme Z may play complementary roles in the cytotoxic immune response. If expressed, granzyme Z-like might modulate the activity of granzyme Z by sequestering its substrates or inhibitors. Furthermore, it has been suggested that some of these granzymes absent in birds induce cell death by mitochondrial or autophagy-related pathways [16].

On the other hand, human cysteine protease paracaspase (*MALT1*) has been shown to modulate T-cell activation [17]. Our analysis has uncovered the zebra finch and chicken orthologues of *MALT1*, along with two additional paracaspase-like genes in zebra finch and chicken, which are not present in human. The phylogenetic analysis of caspases suggests that the common ancestor to mammals and birds had three copies of *MALT1*, two of which were lost in mammals (Figure 1). This result is supported by the finding of three *MALT1-like *genes in *Tetraodon nigroviridis *(CAG08114.1, CAG07960.1, and CAG13116.1).

Taken together, these results suggest that the mammalian granzyme clusters constitute an evolutionary response to special immune challenges, and that the cytotoxic immune response in humans proceeds through more diverse cellular pathways than the corresponding response in birds. In contrast, immune cell activation dependent on paracaspases, might involve more proteases in birds than in mammals.

### Teeth formation

Absence of teeth is a specific feature of birds and adult monotremes. The analysis of avian degradomes shows that both zebra finch and chicken lack two protease genes involved in teeth formation, namely the genes encoding enamelysin (*MMP20*) and kallikrein-4 (*KLK4*) [18-20]. While *KLK4 *is absent in both bird genomes, along with the rest of the kallikrein family of serine proteases, a pseudogene resembling *MMP20 *can be found in a syntenic locus in the chicken genome, but not in the zebra finch genome. Both proteases cleave several enamel proteins necessary for enamel formation in all dentate vertebrates.

Likewise, orthologues of human enamel matrix protein genes (*AMBN*, *AMELX*, and *ENAM*) are absent from avian genomes. In contrast, other non-dentate vertebrates, like platypus, contain orthologues of these genes. This may reflect the fact that, unlike birds, young monotremes display rudimentary teeth. Other genes involved in teeth and bone development are also absent in chicken and zebra finch genomes, including the genes encoding matrix extracellular phosphoprotein (*MEPE*) and dentine-sialophosphoprotein (*DSPP*). Therefore, teeth loss in birds seems to have proceeded through multiple gene losses, including proteases and protease substrates.

### Reproduction

One of the most dissimilar processes between birds and mammals is reproduction. Consistent with this, we have found multiple differences in protease genes involved in fecundation and embryo hatching. Thus, no orthologues of testin serine proteases were found in chicken or zebra finch. In mammals, testins have been linked to spermatogenesis based on localization studies [21]. Testin genes have followed diverse evolutionary patterns, with pseudogenization events in some primates and rodents [4-6]. This suggests that testins might be related to reproductive fitness or even to speciation specifically in mammals. Likewise, several members of the ADAM family of metalloproteases involved in fertilization in mammals are lacking in the genomes of both birds. These include *ADAMs 1-7 *and *ADAM30*. In contrast, other members of this family not involved in reproductive processes are conserved. Notably, a family of two pregnancy-associated plasma metalloproteases, pappalysin-1 and -2 (*PAPPA1 *and *PAPPA2*), seem to be perfectly conserved in birds. Pappalysins are known to cleave IGF-binding proteins (IGFBPs), and overexpression of *PAPPA2 *is related to severe preeclampsia in humans [22]. This result suggests that IGFBP proteases may play a conserved role in pregnancy both in birds and mammals.

**Figure 3:**
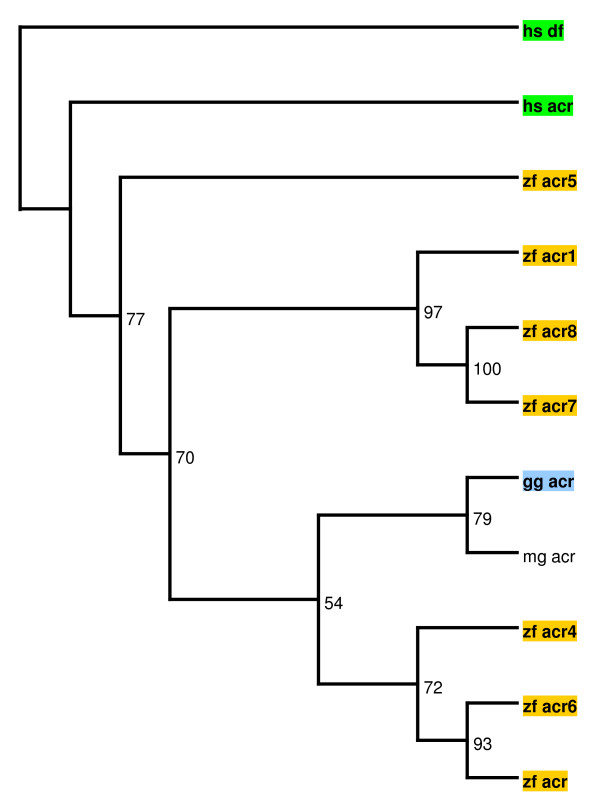
**Phylogenetic relationship between zebra finch (*zf_*), chicken (*gg_*), and human (*hs_*) acrosins**. Numbers indicate the robustness of each node assessed by bootstrap. Acrosin from turkey (*mg_*) is included. Human complement factor (*df*) was included as an outgoup.

On the other hand, acrosin, a serine protease located in the sperm acrosome and involved in the lysis of the zona pellucida to facilitate sperm penetration in the ovum, is conserved in birds and mammals [23]. Strikingly, while the genomes of humans and chickens contain a single acrosin gene (*ACR*), the zebra finch genome contains 7 non-clustered *ACR-like *genes. These include a non-peptidase homolog, named *ACR1*, featuring mutations in all three catalytic residues. Since no orthologues of these novel genes have been found in other organisms, *ACR-like *genes are likely to be due to zebra finch-specific genomic expansions. As shown in Figure 3, some of these zebra finch-specific acrosin-like genes, especially *ACR5*, seem to have undergone non-neutral evolution, likely reflecting reproductive pressures. However, we cannot rule out the possibility of an ancestral genomic expansion of *ACR *followed by loss of the novel genes in several species. The substrates of acrosin are called zona pellucida proteins or ZPs. The human genome encodes four ZP proteins, whereas the chicken genome encodes six ZP family members and the zebra finch genome seven ZPs, due to the specific duplication of *ZPAX *[24].

Consistent with the important differences in reproduction between birds and mammals after fertilization, proteases involved in implantation in mammals and hatching are not conserved. Thus, we have found no evidence of zebra finch or chicken proteases related to murine implantation serine proteases (*ISPs*), which have been shown to participate in embryo implantation and may be involved in embryo hatching [25]. Furthermore, the gene encoding mammalian ovastacin, a metalloprotease which may be involved in embryo hatching [26], is absent in zebra finch and chicken. In contrast, we have found two zebra finch proteases related to fish choryolytic enzymes, which may fulfill this role in embryo hatching. Finally, our analysis has revealed features specific of eutherian organisms. Thus, the alpha-aspartyl dipeptidase gene (*PEPE*), conserved in bacteria, vertebrates, and invertebrates, is present both in zebra finch and chicken, but not in eutherians. Likewise, the gene encoding the aspartyl protease known as nothepsin (*NOTS*) is present in both birds as well as in fish, reptilia and platypus but has been pseudogenized in metatherians and eutherians. Interestingly, both *PEPE *and *NOTS *are highly expressed in reproductive organs of different species. This suggests that the inactivation of these genes, with the concomitant expansion of other protease families, may have played a role in the striking reproductive differences between oviparous and viviparous species.

Taken together, these data show that the profound differences in the reproductive function between birds and mammals are related to multiple gene gain and loss events. Our results suggest that the evolutionary pressure on the reproductive function has driven the expansion of the acrosin family of serine proteases in zebra finch, but not in chicken or mammals. This abrupt change in genes involved in reproduction could potentially lead to reproductive barriers and thus play an important role in speciation.

### Neural development

The zebra finch is an interesting model of neural development. As noted above, the zebra finch genome features a zebra finch-specific *CASP3 *duplication. This event may have important consequences in neural development regardless of the role of this protease in apoptosis. In fact, recent studies have shown that zebra finch *CASP3 *plays a dynamic role in song-response habituation, and therefore is involved in learning and memory [27]. It is important to notice that this result requires experimental validation, since tandem duplication can be mimicked by artifacts in the genomic assembly. Notably, we have found embryo EST sequences corresponding to both *CASP3 *copies. On the other hand, an independent *CASP3 *duplication has been shown in opossum [12].

Additionally, we have found a zebra finch-specific tandem duplication of the gene encoding the aspartyl protease β-secretase 1 (*BACE*). Strikingly, both *CASP3 *and *BACE *have been shown to play a role in αβ peptide accumulation in Alzheimer disease [28]. Since this role is related to β-secretase, it is tempting to speculate that β-secretase activity and regulation in birds may be influenced by these novel copies of *CASP3 *and *BACE*.

Finally, our analysis suggests that the gene encoding the serine protease neurotrypsin (*PRSS12*) was duplicated in an ancestor of birds and mammals, and then one copy was lost in the mammalian lineage (data not shown). Neurotrypsin has been linked to neural development in multiple organisms. Thus, a 4 bp deletion in human *PRSS12 *mRNA is believed to cause mental retardation [29]. Likewise, a *Drosophila melanogaster *strain lacking the orthologue of neurotrypsin has been shown to suffer a long-term memory formation defect [30].

Collectively, these results show several putative duplication events in protease genes involved in neural development. This suggests that gene gain by genomic duplication may underlie some of the differences in neural development between zebra finch and chicken.

### Differences in other proteases

Additional features of the zebra finch degradome may provide clues about the evolution of the zebra finch genome compared to other birds and mammals. Thus, the human genome encodes 24 matrix metalloprotease (*MMP*) genes, involved in multiple biological processes, including development and tissue remodeling [31]. Inspection of the chicken and zebra finch genomes shows that the number of MMPs is much lower, with 16 members each, due to the lack of *MMP-7*, *-8*, *-19*, *-20*, *-21*, *-23B*, *-25*, and *-26*. These dissimilar *MMP *gene sets might underlie some of the differences in bone and cartilage biology or in other tissue-remodelling events between birds and mammals.

Furthermore, while most of the genes in the ADAMTS metalloprotease family are perfectly conserved between zebra finch, chicken, and human, *ADAMTS13 *seems to have been specifically duplicated in zebra finch. It is remarkable that this gene has been related to a human disease called thrombotic thrombocytopenic purpura [32]. This disease causes hemolytic anemia with fragmentation of erythrocytes, thrombocytopenia, diffuse and nonfocal neurologic findings, and decreased renal function. Notably, two members of this family present in humans are lacking in birds. These are *ADAMTS16*, expressed in pre-ovulatory ovarian follicules [33] and linked to inherited hypertension [34], and *ADAMTS4*, which encodes a protease involved in aggrecan degradation and may play a role in the development of osteoarthritis [35]. This suggests that, in birds, other aggrecanases, like ADAMTS-5, fulfill the tasks that ADAMTS-4 performs in mammals.

Finally, the differences in the diets of birds and mammals seem to have prompted the remodelling of the pepsinogen system. Thus, while the human genome contains three pepsinogen A (*PGA*) genes in tandem, birds possess two *PGA *genes duplicated independently. Furthermore, while the chicken genome contains an ortholog of human pepsinogen C (*PGC*), the zebra finch genome seems to be lacking this gene.

We have also found evidence for changes in multiple protease genes which are currently being validated (Table 1). Our ongoing studies on these genes may extend the list of differences in the degradomes of zebra finch, chicken, and humans.

**Table 1:** Comparison of the zebra finch, chicken, human, and mouse degradomes

Gene	Zebra finch	Chicken	Human	Mouse
**Apoptosis**				
Caspase-1, -4, -5, -12	Not found	Only caspase-1	All	All
Caspase-17	Not found	Present	Absent	Absent
Caspase-18	Present	Present	Absent	Absent
**Host defense**				
Granzyme K	Absent	Absent	Present	Present
Neutrophil elastase	Absent	Absent	Present	Present
Complement factor D	Absent	Absent	Present	Present
Azurocidin	Absent	Absent	Present	Absent
Proteinase 3	Absent	Absent	Present	Present
Granzymes B and H, cathepsin G, chymase	Granzyme Z and Z-like	Granzyme Z	All	All
PRSS33	Absent	Absent	Present	Present
Tryptases	Absent	Absent	Present	Present
Haptoglobins	Absent	Absent	Two	One
Cathepsin F	Absent	Absent	Present	Present
Cathepsin W	Absent	Absent	Present	Present
PRSS16	Absent	Absent	Present	Present
Paracaspase	Triplicated	Triplicated	Present	Present
Legumain-2	Absent	Absent	Present	Present
**Tissue development**				
Enamelysin	Absent	Absent	Present	Present
Kallikrein-4	Absent	Absent	Present	Present
MMP-7, -8, -19, -21, -23B, -25	Absent	Absent	Present	Present
MMP-26	Absent	Absent	Present	Absent
ADAMTS-4	Absent	Absent	Present	Present
**Reproduction**				
Alpha-aspartyl dipeptidase	Present	Present	Absent	Absent
Nothepsin	Present	Present	Absent	Absent
ADAMTS-16	Absent	Absent	Present	Present
Testins	Absent	Absent	Present	Present
ADAM3B, -4,-4B,-5,-6	Absent	Absent	Pseudogenes	Present
ADAM7	Absent	Absent	Present	Present
ADAM30	Absent	Absent	Present	Present
Acrosin	Expanded	Present	Present	Present
Prolactin-induced protein	Absent	Absent	Present	Present
Ovastacin	Absent	Absent	Present	Present
ISP1	Absent	Absent	Absent	Present
ISP2	Absent	Absent	Pseudogene	Present
Choryolytic enzymes	Present	Present	Absent	Absent
**Neural development**				
Caspase-3	Duplicated	Present	Present	Present
Neurotrypsin	Duplicated	Duplicated	Present	Present
Bace	Duplicated	Present	Present	Present
Presenilin homolog-2	Absent	Absent	Present	Present
Transmembrane serine protease 5	Absent	Present	Present	Present
Brain serine protease-2	Absent	Absent	Present	Present
**Other**				
Pepsinogen A	Duplicated	Duplicated	Triplicated	Present
Pepsinogen C	Not found	Present	Present	Present
ADAMTS-13	Duplicated	Present	Present	Present
Tubulointerstitial nephritis antigen-like 1	Absent	Absent	Present	Present
Desert hedgehog protein	Absent	Absent	Present	Present
Sentrin-3	Absent	Absent	Present	Present
Autophagin-4	Absent	Absent	Present	Present
NAALADASE like 1	Absent	Absent	Present	Present
Transferrin receptor 2	Absent	Absent	Present	Present
Dipeptidyl-peptidase 3	Absent	Absent	Present	Present
Vitellogenic-like carboxypeptidase	Absent	Absent	Present	Present
ClpP caseinolytic peptidase	Absent	Absent	Present	Present
Abhydrolase domain containing 4	Absent	Absent	Present	Present
Polyserases	One	One	Three	Three
Kallikreins	Absent	Absent	Present	Present
Ubiquitin-specific protease-11	Absent	Absent	Present	Present

## Conclusions

In summary, the analysis of the zebra finch degradome and its comparison with those of other birds and mammals may prove useful in the identification of molecular mechanisms which underlie physiological differences between these organisms. Thus, we have identified changes in protease-coding genes such as caspases, granzymes, acrosins, metalloproteases, and pepsinogens, which might underlie differences in apoptosis, immune system, bone and teeth development, and reproduction. This approach has also allowed us to generate hypothesis about the role of several proteolytic systems in the striking differences in neural development between zebra finch and other birds. In this regard, we have found zebra finch-specific duplications in protease genes involved in neural development, namely *CASP3 *and *BACE*. We expect that these results may contribute to our better understanding of avian biology and zebra finch characteristic traits.

## Methods

### Identification and annotation of zebra finch proteases

To annotate the set of zebra finch proteases, we downloaded the zebra finch genomic sequence deposited in the Genome Center at Washington University, in the context of the Zebra Finch Sequencing and Analysis Consortium (Zebra Finch Sequencing and Analysis Consortium: The genome sequence of the vocal learning zebra finch, submitted). Protease sequences were found and curated with the BATI algorithm (Blast, Annotate, Tune, Iterate), using four in-house Perl scripts: Tbex, BlastSniffer, GeneTuner, and BGmix http://degradome.uniovi.es/downloads. We started with a previously assembled set including curated human [6] and chicken http://www.ensembl.org protease sequences. Each protein sequence in this starting set was compared to the genomic sequence of zebra finch using the TBLASTN program of the BLAST suite [36] with Tbex. Putative orthologues of the starting genes were located with BlastSniffer and curated with GeneTuner. To minimize the number of missed protease genes, a composite file with all of the TBLASTN hits sorted by chromosomal location was also generated with BGmix and inspected. Prediction of chicken and human orthologues was performed by reciprocal best hit analysis. Information on the degradomes of other mammalian species was retrieved from the Degradome database http://degradome.uniovi.es.

### Phylogenetic analysis

In those cases where orthology could not be established by reciprocal best hit analysis, phylogenetic studies were conducted. Protein sequences were aligned with ClustalX [37] and manually edited with Genedoc http://www.nrbsc.org/gfx/genedoc/index.html. Then, alignments were bootstrapped 100 times with Seqboot and most parsimonious trees were generated with Protpars, both from the Phylip package http://evolution.genetics.washington.edu/phylip.html. Trees were displayed with TreeView [37]. Only nodes present in more than 50 bootstrapped trees were displayed.

## Authors' contributions

VQ participated in the study design, performed the annotation process and contributed to write the manuscript; GV participated in the validation of the results; XSP participated in the curation of the initial sequence set; WW participated in the design of the study and CLO participated in the design and coordination of the work and helped to draft the manuscript. All authors read and approved the final manuscript.
